# Joint parameter estimation and abrupt change quantification with uncertainty quantification in high degree of freedom dynamical systems via an optimized adaptive unscented Kalman filter

**DOI:** 10.1007/s44245-026-00298-5

**Published:** 2026-08-03

**Authors:** Esmaeil Ghorbani, Quentin Dollon, Frederick P. Gosselin

**Affiliations:** 1 Department of Civil and Environmental Engineering, Princeton University, 54 Olden Street, Princeton, NJ 08544 USA; 2 Laboratory for Multiscale Mechanics (LM2), Department of Mechanical Engineering, Polytechnique Montréal, A-115.2, 2500, chemin de Polytechnique, Montréal, QC H3T 1J4 Canada; 3 Hydro-Quebec Research Institutes (IREQ), 1800 Bd Lionel-Boulet, Varennes, QC J3X 1S1 Canada

**Keywords:** Unscented Kalman filter, Parameter estimation, Physics-aware optimization, Uncertainty quantification, Partial observation, Damage quantification

## Abstract

The Unscented Kalman Filter (UKF) represents a robust method for estimating latent states and parameters within specified nonlinear equations of dynamic systems under noisy sensor data. Nonetheless, adjusting the filter’s hyperparameters (HP)s is essential for effective performance and poses difficulties, especially in systems with many parameters and states to identify, or when it is necessary for the filter to both detect and measure anomaly levels in parameters. Building on the authors previous research on introducing a physics-aware objective function to tune the UKF, this study advances the capabilities of the objective function to tune different adaptive variants of the UKF which facilitates virtual sensing, joint state-parameter estimation, damage quantification and uncertainty quantification under partial observation for systems with many degrees of freedom (DoF) as an open-ended question in structural health monitoring field. To validate the framework, a three DoF damped mass-spring system experiencing a sudden change in physical characteristics is used. Subsequently, the filter’s precision in estimating parameters and states is evaluated using a ten DoF system with 40 states and unknown parameters, featuring sparsely placed sensors. Furthermore, the Lorenz attractor under partial observation is used as another case study to highlight why and how the physics-aware objective outperforms other commonly used data-driven objective functions. These results demonstrate the potential of the proposed framework for addressing challenging identification problems in dynamical systems such as tracking sudden changes and evaluating the uncertainties linked to both modeling and measurement, particularly those with limited and noisy sensor data.

## Introduction

The Kalman filter (KF) has become a key tool in engineering since the 1960 s [1]. It was first known for its use in aerospace, such as guiding the spacecraft of the Apollo missions [2]. It has since been used in the identification of structural and mechanical systems [3–5], and recently in digital twin technologies to build expressive models and predict the behavior of mechanical and structural systems [6, 7]. Like any inverse problem, the performance of Kalman filtering to accomplish these tasks relies not only on the quality of observed data and a detailed physical model that accurately represents the system’s dynamics, but also on selecting the correct HPs, a process known as filter tuning. This study aims to address this challenging and often time-consuming task that requires careful analysis and expertise, while manual tuning may not be successful at all for complex systems [8].

Different types of KFs have been developed widely applied to various system identification tasks in structural dynamics, including joint state-parameter estimation, damage detection, localization, and quantification, both under routine operating conditions and during extreme events such as earthquakes, as well as in scenarios with partial observations. Although state estimation in systems with many DoFs using KFs is relatively mature, reliable parameter estimation, especially in structural models with many DoFs, remains challenging. This difficulty primarily arises due to the none-identifiably of the system and also sensitivity of filter tuning, particularly regarding model and measurement uncertainties. For example, Wang et al. [9] developed a localized distributed KF to address scalability; however, its HP tuning remains decentralized and intricate. Similarly, Kitanidis [10] introduced a compressed-state KF tailored for low-rank systems but noted persistent challenges in data-dependent model reduction and calibration. Even advanced filtering approaches, such as the iterated Cubature KF explored by Ghorbani and Cha [11], demonstrate accurate state tracking capabilities in high-DoF structural systems but fall short in reliably estimating parameters, due to tuning complexities. Thus, joint state–parameter estimation in systems with many DoFs continues to present significant hurdles related to nonlinearity, high dimensionality, and time-consuming and sometimes impossible HP optimization.

KFs struggle to detect and quantify abrupt parameter changes primarily because they rely on smooth state-transition models and incremental updates, which tend to smooth out sudden discrepancies between predictions and observations. Therefore, KF–based methods are often combined with complementary techniques to effectively detect, localize, and quantify damage. For instance, Ginsberg et al. [12] introduced a sparsity-constrained EKF with $\mathcal {L}^1$ regularization, improving localization but facing scalability limits. Döhler et al. [13] used a predictor-based subspace residual to enhance sensitivity, while Beygzadeh et al. [14] combined filtering with sensitivity analysis, increasing precision at the cost of a high computational time. Yi et al. [15] proposed a minimax-based adaptive filter for uncertain models, and Hoda et al. [16] developed a hybrid Particle-KF for high-resolution crack detection—both improving accuracy, but still limited by complexity and noise sensitivity. Across these studies, filter tuning remains a major challenge, and adding sensitivity terms introduces additional HPs, which intensifies with system size and hinders scalability.

Many studies have focused on tuning the HPs of the KF to facilitate the above-mentioned identification tasks. Most existing approaches formulate this task as an optimization or statistical estimation problem aimed at improving consistency with sensor measurements [17, 18]. Different criteria have been proposed, including maximum likelihood and Bayesian inference [19], least-squares fitting after Kalman smoothing [20], auto-/cross-correlation analysis and covariance identification [21], innovation-based adaptive estimation, covariance matching, and expectation-maximization-based estimation of modeling error (*Q*) and mesurement error (*R*) covariance matrices. For nonlinear filters, recent studies have also treated UKF tuning as a black-box optimization or learning problem. For example, [22] used Bayesian optimization with a Student-*t* process, while [23] employed a recurrent neural network to adjust UKF HPs. Despite differences in optimization strategy, these methods remain primarily data-consistency-driven because the filter is tuned using measurement residuals, innovation statistics, likelihood functions, or output-prediction errors.

In parallel, physics-informed and physics-guided estimation methods have shown that embedding governing equations or physical constraints can improve robustness under sparse, noisy, or partially observed data [24–27]. However, most existing physics-informed filtering studies either modify the state-transition model, introduce physics-guided surrogate models, impose constraints during the filtering update, or assimilate data using a fixed or separately tuned filter. They do not directly address the problem considered here: automated outer-loop optimization of Kalman filter HPs using the residual of the governing physical equations evaluated from the estimated states and parameters.

Although prior studies have proposed various optimization techniques for tuning HPs, their fundamental objective commonly remains tied to data-based criteria, such as the MSE between sensor measurements and model-predicted outputs. This strategy can be redundant and ineffective because the KF is already structured to reduce measurement-prediction mismatch internally through the correction step. Moreover, accurate output tracking does not necessarily imply accurate latent-state or physical-parameter identification. This limitation becomes particularly important in challenging scenarios such as sparse sensor data [28], systems with many DoFs [29], abrupt physical changes [30], and noisy or partially observed measurements. In contrast, the proposed framework uses the residual of the governing equations as the outer-loop objective for HP optimization, thereby encouraging the filter to produce estimates that are not only measurement-consistent but also dynamically admissible.

To optimize HPs without using sensor data in the objective function, we have previously [31] defined a new *physics-aware objective function* that uses the states and parameters estimated by the KFs. This leads to a hands-free automated framework that tunes the HPs of a KF for state-parameter identification tasks in different systems in various engineering fields. The physics-aware objective function focuses on tuning the HPs by minimizing the residual of the physics equations instead of just matching the estimated state corresponding to sensor data. This strategy is particularly effective when dealing with partial observations and provides a solid foundation when the physics of the system is well understood. Its validity has since been further demonstrated on experimental data in a follow-up study on hydrodynamic bearings [32], confirming the robustness of the physics-aware loss beyond purely numerical tests. The idea involves adjusting HPs in an external optimization loop, using a nonlinear mesh adaptive direct search algorithm (NOMAD) [33] to minimize the physics-aware objective function.

In this study, we propose to extend upon the work of reference [31] by rigorously evaluating and pushing the limits of the physics-aware objective function to enhance the UKF performance in different challenging system identification scenarios. Now, building on the demonstrated validity of the loss—including experimental verification in [32, 34]—we move one step forward to showcase new capabilities and broader applicability of this framework. Specifically, we focus on challenging tasks such as joint state-parameter estimation for systems with many DoFs, abrupt change detection and quantification, and partial observation conditions–challenges that have not been comprehensively addressed before. We chose the UKF because it is known for its accurate and near real-time performance in estimating the parameters and states of any dynamical system as a probabilistic inference method [35, 36]. However, this filter needs HP tuning to ensure proper performance similarly to any other type of Bayesian filtering method. Incorrect settings can lead to filter divergence or poor estimation performance [37].

After a brief overview of the theoretical background in Sect. 2, we present the physics-aware optimization framework in Sect. 3 and then investigate three case studies. First, in Sect. 4.1 we examine the robustness of the optimal HPs when tracking a damped mass-spring system that undergoes sudden parameter changes. In Sect. 4.2 we show how the physics-aware loss helps to find the UKF optimal HPs to estimate the stiffness and damping matrices of a ten-DoF system with 40 unknown states and parameters under sparse and noisy sensor data. Third, in Sect. 4.3, we use Lorenz attractor as a case study to compare the effectiveness of the physics-aware objective function against other commonly used objective functions, demonstrating why the physics-aware approach is superior for HP optimization for joint state and parameter estimation of a chaotic system under partial observations and noisy data.

## Unscented Kalman filter

Consider the general form of a noisy, discrete-time state-space representation of a dynamical system

1 $$\begin{aligned} \mathbf{x}_{k+1} = \mathcal {M}(\mathbf{x}_k, \mathbf{u}_k, \boldsymbol{\theta }) + \boldsymbol{\nu }_k, \quad \quad \boldsymbol{\nu }_k \sim \mathcal {N}(0, \mathbf{Q}), \end{aligned}$$

where $\mathbf{x}_{k}$ denotes the state variable, $\mathbf{u}_k$ represents the input term and $\boldsymbol{\theta }$ refers to the system parameters at time step *k*. The transition function $\mathcal {M}$ predicts the state variable $\mathbf{x}_{k+1}$ in the next time step incorporating the modeling error $\boldsymbol{\nu }_k$, which follows a Gaussian distribution with mean zero and covariance $\mathbf{Q}$. The predicted state variable at any time step can be used to predict the system output $\hat{\mathbf{y}}_k$ at the same time step as,

2 $$\begin{aligned} \hat{\mathbf{y}}_k = \mathcal {H}(\mathbf{x}_{k}, \mathbf{u}_k, \boldsymbol{\theta }) + \boldsymbol{\mu }_k, \quad \quad \boldsymbol{\mu }_k \sim \mathcal {N}(0, \mathbf{R}), \end{aligned}$$

in which $\mathcal {H}$ is the measurement function, and $\boldsymbol{\mu }_k$ describes the zero mean measurement error with covariance $\mathbf{R}$.

The UKF method, similar to any type of Kalman filtering method, compares the predicted output $\hat{\mathbf{y}}_k$ at any time step *k* with the corresponding measurement $\mathcal {Y}_{1:N}=\{\mathbf{y}_1,\ldots ,\mathbf{y}_N\}$. The filter estimates the states and identifies the parameters of the underlying system by minimizing the mean squared error between the predicted output and the measured data.

Considering the discrete state-space form of the dynamical system and knowing the forcing term ($\mathbf{u}$) at each time step, to start the UKF algorithm, we first rearrange all states and parameters in an augmented state vector $\mathbf{x}^a=[\mathbf{x},\boldsymbol{\theta }]$ to build an augmented transition function $\mathbf{x}^a_{k+1} = \mathcal {M}^a(\mathbf{x}^a_k, \mathbf{u}_k)$. Afterward, assuming that the desired states and parameters have a Gaussian distribution, we assign an uncertain initial value to this distribution with mean $\mathbf{x}^a_0$ and covariance $\mathbf{P}^a_0$ as our prior knowledge of the system. The initial mean and covariance are passed into a nonlinear transformation named unscented transform (UT):

3 $$\begin{aligned} \begin{array}{lcl} {\chi }^i & =& \mathbf{x}_k^a, \quad \quad \quad \quad \quad \quad \quad \quad i = 0,\\ {\chi }^i & =& \mathbf{x}_k^a + \sqrt{(n + \lambda ) {\mathbf{P}}_{[i]}^a}, \quad i = 1,2, \ldots , n, \\ {\chi }^i & =& \mathbf{x}_k^a - \sqrt{(n + \lambda ) {\mathbf{P}}_{[i]}^a}, \quad i = n+1, \ldots , 2n, \end{array} \quad \begin{array}{lcl} w_m^0 & =& \frac{\lambda }{n+\lambda }, \\ w_c^0 & =& \frac{\lambda }{n+\lambda }, \\ w_m^i & =& w_c^i = \frac{1}{2(n+\lambda )}, \quad i = 1, \ldots , 2n, \end{array} \end{aligned}$$

which generate *2n+1* weighted points named sigma points (denoted as ${\chi }$ and $w_m$, $w_c$) in an *n* dimensional space where *n* is the length of the augmented state vector. The scaling factor *λ *, as an HP, defines the spread of the sigma points around the mean vector, while $w_m$ and $w_c$ represent the weights used to calculate the predicted mean of the states and the weighted covariance matrix, respectively. The term ${\mathbf{P}}_{[i]}^a$ corresponds to the *i*th column of the augmented covariance matrix [11]. These auxiliary points (Eq. 3) help approximate the mean and covariance of the predicted states and outputs propagated through the nonlinear transition and measurement functions rather than linearizing them to be used in an extended KF or a linear KF [38].

In each time step, the sigma points are passed through the augmented transition function $\mathcal {M}^a$ to obtain the transformed sigma points and evaluate the sample mean.

4 $$\begin{aligned} {\left\{ \begin{array}{ll} \mathbf{X}_{k+1|k}^i = \mathcal {M}^a ({\chi }_k^i, \mathbf{u}_k), \\ \bar{\mathbf{X}}_{k+1|k}=\sum _{i=0}^{2n} w_m^i \mathbf{X}_{k+1|k}^i. \end{array}\right. } \end{aligned}$$

Considering the zero-mean modeling error in the transition function, the sigma points and the corresponding sample mean are used to calculate the states covariance matrix as:

5 $$\begin{aligned} {\mathbf{P}}_{k+1}^{XX} = \sum _{i=0}^{2n} w_c^i [ \mathbf {{X}}_{k+1|k}^i - \bar{\mathbf{X}}_{k+1|k}] [\mathbf {{X}}_{k+1|k}^i - \bar{\mathbf{X}}_{k+1|k}]^ \top + {\mathbf{Q}}. \end{aligned}$$

In a similar vein, the transformed sigma points are propagated into the measurement function $\mathcal {H}$, and the mean of the sample output is calculated for each time step.

6 $$\begin{aligned} {\left\{ \begin{array}{ll} \hat{\mathbf{Y}}_{k+1|k}^i = \mathcal {H}(\mathbf{X}_{k+1|k}^i, \mathbf{u}_k),\\ \bar{\mathbf{Y}}_{k+1|k}=\sum _{i=0}^{2n} w_m^i \hat{\mathbf{Y}}_{k+1|k}^i. \end{array}\right. } \end{aligned}$$

The predicted output covariance matrix is calculated using the following equation considering the measurement error (${\mathbf{R}}$):

7 $$\begin{aligned} {\mathbf{P}}_{k+1}^{YY} = \sum _{i=0}^{2n} w_c^i [ \hat{\mathbf{Y}}_{k+1|k}^i - \bar{\mathbf{Y}}_{k+1|k}] [ \hat{\mathbf{Y}}_{k+1|k}^i - \bar{\mathbf{Y}}_{k+1|k}]^\top + {\mathbf{R}}. \end{aligned}$$

The cross covariance matrix is also calculated as follows:

8 $$\begin{aligned}{\mathbf{P}}_{k+1}^{XY}&= \sum _{i=0}^{2n} w_c^i[\mathbf{X}_{k+1|k}^i - \bar{\mathbf{X}}_{k+1|k}][ \hat{\mathbf{Y}}_{k+1|k}^i - \bar{\mathbf{Y}}_{k+1|k}]^\top .\end{aligned}$$

The cross-covariance between the predicted states and the measurement vectors normalized by the predicted measurement covariance defines the Kalman gain ($\mathcal {K}$) as:

9 $$\begin{aligned} \mathcal {K}_{k+1}={\mathbf{P}}_{k+1}^{XY} ({\mathbf{P}}_{k+1}^{YY})^{-1}, \end{aligned}$$

that functions as a regulator to update the predicted state mean and covariance as:

10 $$\begin{aligned} &\hat{\mathbf{x}}_{k+1}^a&=\bar{\mathbf{X}}_{k+1|k} +\mathcal {K}_{k+1} (\mathbf{y}_{k+1}-\bar{\mathbf{Y}}_{k+1|k}), \end{aligned}$$

11 $$\begin{aligned} & \mathbf {P_{k+1}^a} ={\mathbf{P}}_{k+1}^{XX} - \mathcal {K}_{k+1} {\mathbf{P}}_{k+1}^{YY} {\mathcal {K}_{k+1}}^\top . \end{aligned}$$

The updated mean $\hat{\mathbf{x}}_{k+1}^a$ and the covariance matrix $\mathbf{P}_{k+1}^{a}$ are sent back to Eq. 3 as an iterative process until the values of the augmented state vector catch the sensor data.

### Adaptive modeling and measurement uncertainty

In the UKF algorithm described above, it is assumed that the modeling and measurement error matrices are defined as $\mathbf{Q} = q^2_0 \mathbf{I}_n$ and $\mathbf{R} = r^2_0 \mathbf{I}_{\hat{n}}$, where $\mathbf{I}$ is the identity matrix while *n* and $\hat{n}$ represent the dimension of the state and output vectors, respectively. The HPs $q_0$ and $r_0$ are arbitrarily chosen constants. We call that filter **UKF-3HP**, because it has three HPs that need to be adjusted (*λ *, $q_0$ and $r_0$). However, in a real scenario, the level of uncertainty is not always constant throughout the identification process [39]. In this study, to improve the performance of the UKF-3HP and also quantify modeling and measurement errors, we consider two different adaptive schemes to update $\mathbf{Q}$ and $\mathbf{R}$ through the UKF iteration.

The first adaptive scheme is the forgetting factor method, introduced as **UKF-F5HP**, that uses the difference between the predicted output and the corresponding sensor data called residual ($\boldsymbol{\varepsilon }$) at each time step

12 $$\begin{aligned} \boldsymbol{\varepsilon }_{k+1} ={\mathbf{y}}_{k+1} -\bar{\mathbf{Y}}_{k+1|k}, \end{aligned}$$

and update the process and measurement covariance matrices using two separate forgetting factors ($\alpha _q$, $\alpha _r$), as HPs:

13 $$\begin{aligned} \mathbf{Q}_{k+1}= (1-\alpha _q) \mathbf{Q}_{k} + \alpha _q (\mathcal {K}_{k+1} \boldsymbol{\varepsilon }_{k+1}) (\mathcal {K}_{k+1} \boldsymbol{\varepsilon }_{k+1})^\top , \end{aligned}$$

14 $$\begin{aligned} \mathbf{R}_{k+1} = (1-\alpha _r) \mathbf{R}_{k} + \alpha _r (\boldsymbol{\varepsilon }_{k+1}\boldsymbol{\varepsilon }_{k+1}^\top + {\mathbf{P}}_{k+1}^{YY}). \end{aligned}$$

**UKF-F5HP** method uses five HPs in the tuning process (*λ *, $q_0$, $r_0$,$\alpha _q$,and $\alpha _r)$. Details of the forgetting factor adaptive procedure are provided in [40].

The second adaptive scheme is the moving-window covariance matching technique which we call it **UKF-W5HP**. This technique is used to update the modeling and measurement uncertainty in each iteration considering a segment of the signal:

15 $$\begin{aligned} \mathbf{Q}_{k+1}=\frac{1}{w_q}\sum _{i=0}^{w_q-1} (\mathcal {K}_{k+1-i}\boldsymbol{\varepsilon }_{k+1-i}) (\mathcal {K}_{k+1-i}\boldsymbol{\varepsilon }_{k+1-i})^\top , \end{aligned}$$

16 $$\begin{aligned} \mathbf{R}_{k+1} = \frac{1}{w_r} \sum _{i=0}^{{w_r}-1} \boldsymbol{\varepsilon }_{k+1-i} \boldsymbol{\varepsilon }_{k+1-i}^\top + {\mathbf{P}}_{k+1}^{YY}. \end{aligned}$$

Equations 15, 16 reveal that a larger window size provides a smoother and slower change in uncertainty, and the algorithm will be less responsive to changes in the system. The moving window technique also brings two HPs ($w_r$, and $w_q$) to the list of HPs in the UKF.

As another adaptive scheme, we integrate both forgetting factor and moving-window techniques together in the UKF which brings seven HPs in total to the optimization problem and we call it **UKF-WF7HP**. It is worth mentioning that both methods, forgetting factor and moving window, are computable surrogates of an intractable asymptotic model called covariance matching, and more details of the UKF and different adaptive covariance calculation techniques are available in [41–43].

## Tuning of the unscented Kalman filter

Developing a robust predictive model becomes increasingly complex in scenarios where sensors are sparsely positioned and the system lacks full observability. In such cases, Kalman filtering serves effectively as a virtual sensor to estimate states and identify parameters within unobserved regions; however, tuning the filter for reliable performance remains a significant challenge [30]. To address this issue, we adopt the offline optimization framework of [31] that integrates the NOMAD black-box optimization algorithm as a meta-optimization tool to determine the optimal HPs for the UKF. The optimization starts with an initial value and range of variation for all HPs to minimize the $L_2$ norm physics-aware objective function:

17 $$\begin{aligned} \mathcal {L}_{\text {physics}}^{2} =\left( \sum _{k=k_1}^{k_2} \left\| \hat{\mathbf{x}}_{k+1}-\mathcal {M}(\hat{\mathbf{x}}_k,\mathbf{u}_k,\hat{\boldsymbol{\theta }}_k)\right\| _2^2 \right) ^{1/2}. \end{aligned}$$

where $k_1$ and $k_2$ define the time window over which the estimated states and parameters are extracted for the loss calculation. Whereas the state variables $x_k, x_{k+1}$ solve Eq. 1, the updated state variables $\hat{x}_k, \hat{x}_{k+1}$ do not necessarily do so. Optimizing the HPs of the UKF to minimize Eq. 17 incentivizes the filter to find the right parameters *θ * rapidly, so that little update to the predicted state is necessary for the predicted output to match the measurement.

To ensure reproducibility and facilitate implementation, Algorithm 1 presents the full pseudocode for our proposed NOMAD-Tuned UKF framework. The algorithm follows the same notation and indexing as the theoretical formulation in Sect.  2. Initially, a set of HPs ($\boldsymbol{\phi }_0$) is assigned, and the filter estimates the states and parameters using the given model and sensor data as input. The estimated states and parameters of the UKF are evaluated using the physics-aware objective function (Eq. 17) within the NOMAD optimizer. The optimizer then iteratively refines the HPs to improve estimation accuracy by minimizing the objective function, progressively converging towards champion HPs ($\boldsymbol{\phi }^*$). The dimension of the optimization space depends on the type of UKF chosen for the identification task. For example, the summary of all UKF variants that we use in this study, the number and type of HPs, is listed in Table 1.

**Table 1:** HP settings for UKF variants based on adaptive scheme

Type of UKF	HPs
UKF-3HP	$(r_0, q_0, \lambda )$
UKF-F5HP	$(r_0, q_0, \alpha _r, \alpha _q, \lambda )$
UKF-W5HP	$(r_0, q_0, w_q, w_r, \lambda$ )
UKF-WF7HP	$(r_0, q_0, \alpha _r, \alpha _q, w_q, w_r, \lambda )$

**Algorithm 1:**
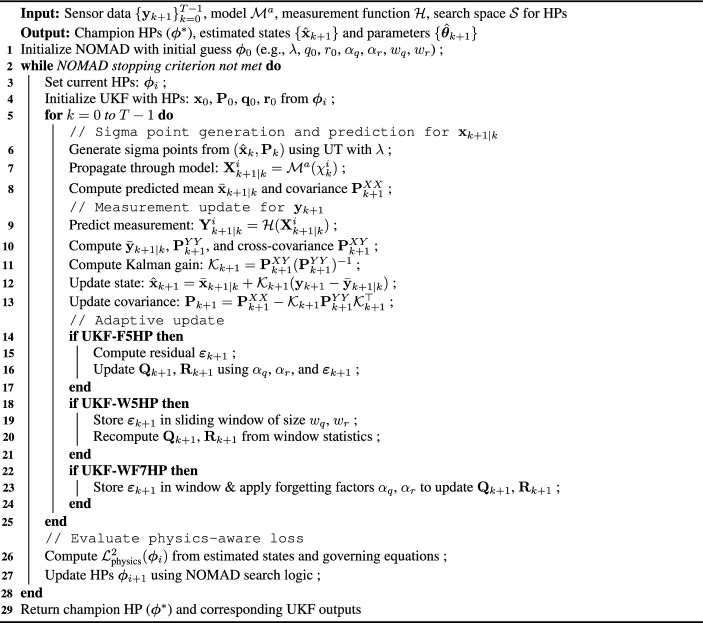
Physics-aware NOMAD-Tuned UKF with Adaptive Covariance Estimation

**Fig. 1:**
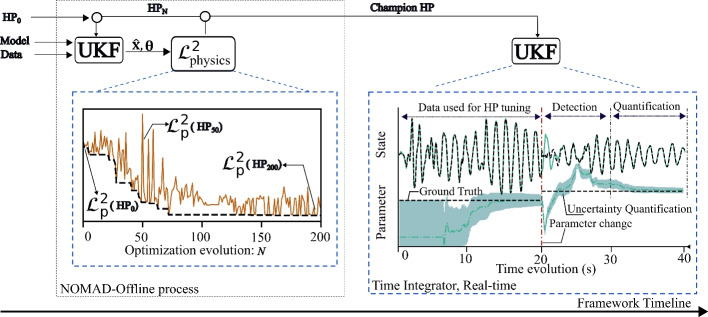
UKF-based HP tuning and identification framework. The process begins with an initial set of HPs for the UKF, which estimates states and parameters using model and sensor data. The estimates are evaluated via the physics-aware objective function $\mathcal {L}^2_{\text {physics}}$ within the NOMAD optimizer, which iteratively updates the HPs to improve estimation accuracy. The left panel shows the offline optimization process, where $\mathcal {L}^2_{\text {physics}}$ decreases as HPs are refined. The right panel illustrates real-time state and parameter estimation using the optimized HPs. The framework enables detection, quantification, and uncertainty estimation, ensuring robust system tracking even under parameter changes

The schematics of the meta-optimization framework is shown in Fig. 1 to faciliate the understanding of the framework. The left panel represents the NOMAD offline process, where the evolution of the objective function over multiple optimization steps (*N*) is shown. The process refines HPs by iteratively reducing the error in joint state and parameter estimation process. As optimization progresses, the $\mathcal {L}^2_{\text {physics}}$ function decreases, indicating better filter performance.

Once the champion set of HPs is determined, it is used by the UKF for online state and parameter estimation, as shown in the right panel. Here, the solver integrates the UKF in time to dynamically estimate the state and parameters of the system. The right panel also shows the effect of a sudden parameter change (marked by the red dashed line) and the corresponding robustness of the optimal HPs to track system behavior. The estimated state and parameters (green line) closely follow the ground truth, while the shaded region represents uncertainty quantification. The optimized HPs ensure that the UKF can effectively detect, quantify, and track the dynamics of the system even under evolving conditions.

We chose NOMAD [33] to optimize the filter because, as a derivative-free optimization methodology, it is specifically designed to tackle the challenges associated with complicated objective functions that may be computationally demanding, noisy, non-convex or inherently complex. Importantly, both the UKF and NOMAD operate without requiring derivative information, making the combined UKF-NOMAD framework particularly well-suited for highly nonlinear systems where analytical gradients are difficult or impossible to obtain, or where their computation could introduce instabilities. This synergy enables robust and efficient hyperparameter tuning even in challenging scenarios with complex dynamics and noisy sensor data. A primary strength of NOMAD is its adaptive mesh refinement mechanism, which intelligently balances broad exploration with detailed exploitation. Another notable aspect of NOMAD is its methodological reliability and structural clarity without the need for interaction with experts. Intrinsic convergence properties, established under certain conditions, provide assurance regarding the method’s trajectory towards optimal or near-optimal solutions [44].

This framework proves especially effective for tuning the filter as virtual sensor in scenarios where only partial observations of the system are available. It leverages a deep understanding of the system’s underlying physics to guide the estimation process, thereby providing a robust and reliable foundation for accurate joint state and parameter estimation even when sensor data is sparse and noisy [32]. Another key advantage of this approach is that, as it avoids directly relying on raw sensor data to determine optimal HP, it mitigates residual fluctuations caused by noisy sensor data and reduces the risk of overfitting during HP tuning. As another advantage, the physics-aware optimized adaptive KFs will quantify both modeling and measurement uncertainties, making it a robust tool for complex systems with limited data.

However, this methodology is not without challenges. For instance, the optimization process becomes more difficult if the underlying state-space model is non-identifiable, potentially leading to unstable or misleading results.

The computational cost of the proposed framework is dominated by repeated UKF evaluations within the NOMAD outer loop. NOMAD is based on mesh adaptive direct search (MADS), a derivative-free black-box optimization strategy designed for problems where objective evaluations may be noisy, nonconvex, nonsmooth, or expensive [33, 45]. This makes NOMAD suitable for the present HP-tuning problem, where analytical gradients of the complete UKF-based objective are unavailable and the loss landscape can be nonconvex. Therefore, the total computational burden depends on both the number of NOMAD objective evaluations and the dimension/type of the selected Kalman filter. For istance, the UKF itself has a known scalability limitation because it propagates *2n+1* sigma points for an augmented state of dimension *n* [34]. Importantly, in the proposed framework, NOMAD is used only for offline HP optimization. Once the champion HPs are obtained, they can be reused for related identification scenarios, as demonstrated by the robustness of the optimized framework under parameter changes and different adaptive UKF variants. Scaling the proposed method to very large-scale systems remains an open question. Future work could investigate online tuning schemes and parallelization strategies to reduce the wall-clock time of the optimization, such as parallel UKF evaluations within NOMAD or parallel NOMAD runs initialized from different HP values.

We used the open-source Python package PyNomadBBO, which provides a Python interface to the NOMAD black-box optimizer. This library allows direct and flexible integration of derivative-free optimization into Python workflows, making it well-suited for tuning KF HPs where the objective function is noisy, nonconvex, or lacks analytical gradients. With minimal setup, users can define their own objective function (e.g., our physics-aware loss in Eq. 17) and pass bounds and initial guesses for each hyperparameter. The library handles constraint enforcement, adaptive mesh refinement, and convergence tracking internally, enabling reproducible and efficient optimization. The package is available at https://pypi.org/project/PyNomadBBO/.

## Case studies

The selected case studies aim to explore the effectiveness of the framework in more depth and demonstrate its applicability in a wider range of previously unexamined scenarios [31]. First, we assess the robustness of the UKF with tuned HPs in tracking abrupt parameter changes indicative of damage. We want to answer the question of whether we need to re-tune the filter once any damage (sudden parameter change) occurred in the system during identification process or not. Secondly, we assess the precision of the framework in optimizing an adaptive UKF for a dynamical system with many DoFs comprising approximately 40 states and parameters, specifically a 10-DoF damped mass-spring system, under conditions of partial observation and noisy data. Finally, we investigate the reasons behind the superior performance of the physics-aware objective compared to the data-based objective.

All simulation data are generated using the Livermore Solver for Ordinary Differential Equations (LSODA) numerical solver [46]. Independent and identically distributed white noise is added to generalize the problems. As the discretization of the transition function depends on the chosen algorithm, for the first and third case studies, the continuous state transition function ($\mathcal {M}_\text {c}$) is introduced in the corresponding section. We have omitted the transition function for the second case study to conserve space, as its derivation is straightforward. In this study, we use an explicit predictor-corrector scheme, also named the Heun method [47], a typical two-stage discretization technique that uses the trapezoidal rule to extend the Euler scheme as follows:

18 $$\begin{aligned} \mathbf{x}^*_{k+1}&= \mathbf{x}_k + \Delta t \, \mathcal {M}_c(\mathbf{x}_k, \mathbf{u}_k, \boldsymbol{\theta }), \\ \mathbf{x}_{k+1}&= \mathbf{x}_k + \dfrac{\Delta t}{2} \left[ \mathcal {M}_c(\mathbf{x}_k, \mathbf{u}_k, \boldsymbol{\theta }) + \mathcal {M}_c(\mathbf{x}^*_{k+1}, \mathbf{u}_{k+1}, \boldsymbol{\theta }) \right] , \end{aligned}$$

where $\mathcal {M}_c(\mathbf{x}_{k}, \mathbf{u}_{k}, \boldsymbol{\theta })$ is the continuous version of the transition function and *Δ t* is the sampling rate.

### Case study 1: tuning hps to quantify the level of damage

In this case study, we first use the framework to find optimal HPs for different variants of adaptive UKFs; and secondly, we evaluate how sensitive the optimized HPs of the UKF are to a sudden parameter change in the system’s physics.

#### Problem formulation

To conduct this analysis, we simulate a three-story structure using a three DoF damped mass-spring system subjected to earthquake excitation. The sensor data consists of noisy vertical acceleration measurements recorded during the earthquake event. The system’s governing equation of motion is written as:

19 $$\begin{aligned} \mathbf{M} \ddot{\mathbf{q}} + \mathbf{C} \dot{\mathbf{q}} +\mathbf{K} \mathbf{q} = -\mathbf{M} \mathbf{a}_{\text {g}}, \end{aligned}$$

in which $\mathbf{q}$ is the vector of the generalized coordinates, each $q_i$ being the displacement at DoF *i*, and following that, the vectors $\dot{\mathbf{q}}$ and $\ddot{\mathbf{q}}$ denote velocity and acceleration, respectively. The matrices $\mathbf{M}$, $\mathbf{C}$, and $\mathbf{K}$ are the mass, damping, and stiffness matrices, and are described as:

20 $$\begin{aligned} \mathbf{M} = \begin{pmatrix} m_1 & 0 & 0 \\ 0 & m_2 & 0 \\ 0 & 0 & m_3 \end{pmatrix},\quad \mathbf{K} = \begin{pmatrix} k_1 + k_2 & -k_2 & 0 \\ -k_2 & k_2 + k_3 & -k_3 \\ 0 & - k_3 & k_3 \end{pmatrix},\quad \mathbf{C} = \begin{pmatrix} c_1 + c_2 & -c_2 & 0 \\ -c_2 & c_2 + c_3 & -c_3 \\ 0 & - c_3 & c_3 \end{pmatrix}. \end{aligned}$$

We define the mass, stiffness, and damping values (in Eq. 20) as $m_i = {500}\,\mathrm{kg}$, $k_i ={50\,000}\,\mathrm{N}/\mathrm{m}\,$, and $c_i = 300\,{\hbox {N}}\,{\hbox {s/m}}$ for all DoF *i=1,2,3*. The system is subjected to the El Centro earthquake acceleration record, denoted as $\mathbf{a}_{\text {g}}$, which serves as ambient excitation [48], and synthetic data, the acceleration of vibration, for each DoF is captured from the simulation results at a sampling rate of 250 Hz over the span of 50 s.

To generalize the identification process and account for measurement uncertainty, white noise with a signal-to-noise ratio (SNR) of 20 is added to the acceleration of vibration used as measurements (sensor data) $\mathbf{y} = (\ddot{\mathbf{q}}_1, \ddot{\mathbf{q}}_2, \ddot{\mathbf{q}}_3)$, where $\ddot{\mathbf{q}}_1$ represents the time history of vibration acceleration for the first DoF, and so on (Eq. 23). The objective is to estimate the displacements ($q_i$), velocities ($\dot{q}_i$), and latent parameters, including damping ($c_i$) and stiffness ($k_i$), for each DoF, both before and after the occurrence of the abrupt parameter change to the system. Consequently, the augmented state vector is defined as follows:

21 $$\begin{aligned} \mathbf{x} &= \left( q_1, q_2, q_3,\; \dot{q}_1, \dot{q}_2, \dot{q}_3,\; k_1, k_2, k_3,\; c_1, c_2, c_3\right) ^\top\\& = \left( x_1, x_2, x_3, x_4, x_5, x_6, x_7, x_8, x_9, x_{10}, x_{11}, x_{12} \right) ^\top . \end{aligned}$$

Following that, the augmented continuous state transition matrix is derived as:

22 $$\begin{aligned} \mathcal {M}_\text {c}^{\text {3DoF}}(\mathbf{x}_k, \mathbf{u}_k) = \begin{bmatrix} x_4 \\ \\ x_5 \\ \\ x_6 \\ \\ -a_{\text {g}} + \frac{-x_7 x_1 - x_8 x_1 + x_8 x_2 - x_{10}{x_4} - x_{11} x_4 + x_{11} x_5}{m_1} \\ \\ -a_{\text {g}} + \frac{x_8 x_1 - x_8{x_2 } - x_9 x_2 + x_9 x_3 + x_{11} x_4 - x_{11}{x_5 } - x_{12} x_5 + x_{12} x_6}{m_2} \\ \\-a_{\text {g}} + \frac{x_9 x_2 + x_{12} x_5 - x_{12} x_6 - x_9 x_3}{m_3} \\ \\ \mathbf{0}_{6\times 1} \\ \end{bmatrix}, \end{aligned}$$

where the subscript $\text {c}$ stands for continuous function. Following that the measurement function $\mathcal {H}^{\text {3DoF}}$ is defined in Eq. 23 as:

23 $$\begin{aligned} \mathcal {H}^{\text {3DoF}}(\mathbf{x}_{k}, \mathbf{u}_k) = \begin{bmatrix} \ddot{q}_1 \\ \\ \ddot{q}_2\\ \\ \ddot{q}_3 \end{bmatrix} = \begin{bmatrix} -a_\text {g} + \frac{-x_7{x_1 } - x_8 x_1 + x_8 x_2 - x_{10}{x_4 } - x_{11} x_4 + x_{11} x_5}{m_1} \\ \\ -a_\text {g} + \frac{x_8 x_1 - x_8{x_2 - x_9 x_2 + x_9 x_3 + x_{11} x_4 - x_{11}{x_5 } - x_{12} x_5 + x_{12} x_6}}{m_2} \\ \\ -a_\text {g} + \frac{x_9 x_2 + x_{12} x_5 - x_{12} x_6 - x_9 x_3}{m_3} \end{bmatrix}. \end{aligned}$$

**Fig. 2:**
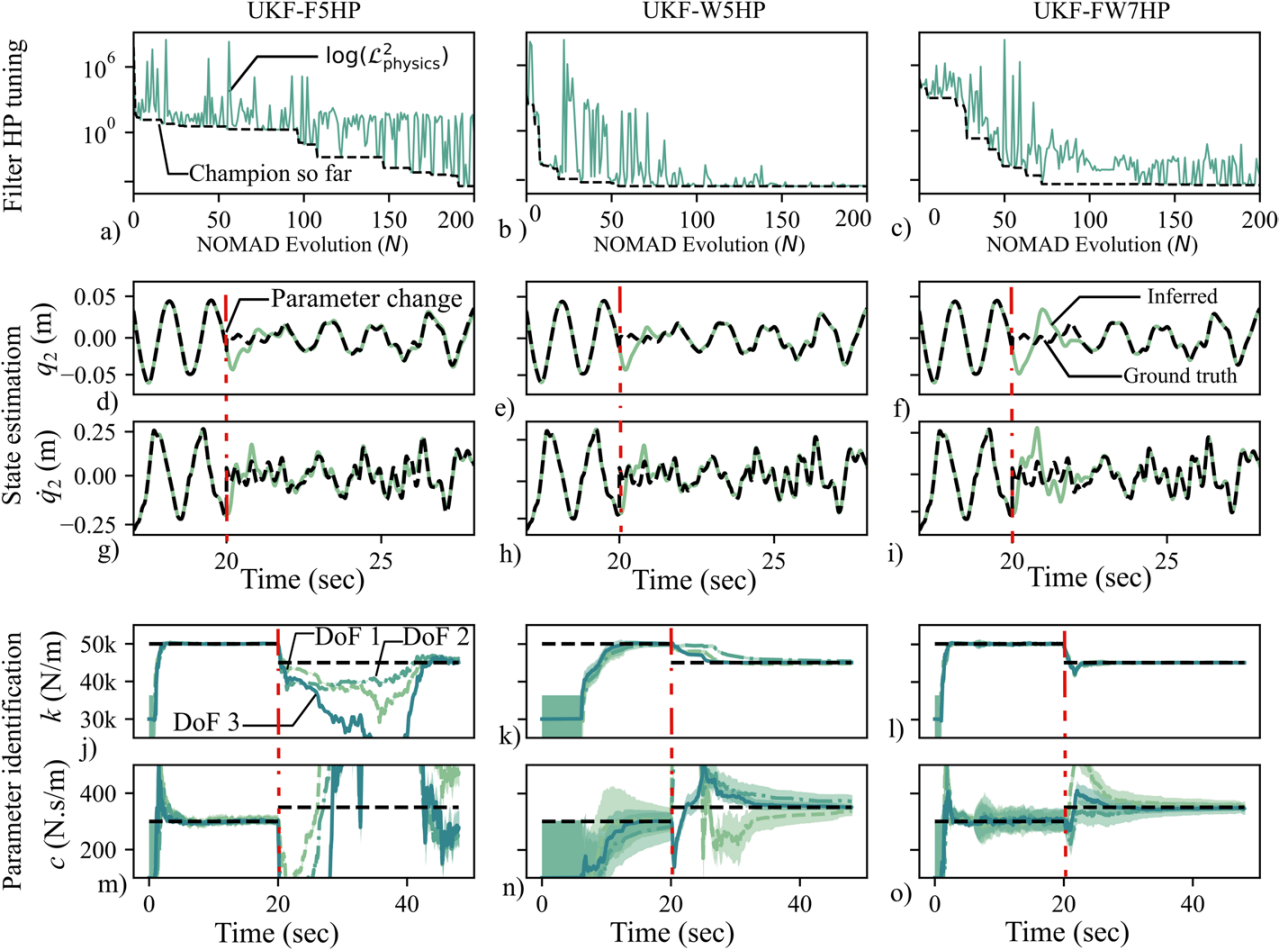
Comparison of the optimized filters performance in capturing the states and parameters before and after the sudden parameter change. Figures **a**–**c** illustrate the evolution of the physics-aware objective function (green line) and the best result achieved through the evolution (black line mentioned as champion so far). Figures **d**–**i** show the estimated states for the second DoF based on the optimal HPs achieved. The red dashed line indicate the parameter change imposed to the system. Figures **j**–**o** illustrate the identified stiffness and damping parameters for each DoF for the different variants of UKF based on the optimal HPs

To estimate the states and identify the parameters, we need to optimize the HPs. Based on the schematic shown in Fig. 1, the initial HPs are set to $(r_0, q_0, \alpha _r, \alpha _q, w_q, w_r, \lambda ) = (0.01, 0.0006, 0.6, 0.6, 5000, 4500, 0)$ for the three variants of the UKF. The optimization process is conducted over 200 iterations and the evolution of the objective functions are shown in Fig. 2 (a, b, and c), to find the best set of HPs for different variants of the UKFs listed in Table 2.

The UKF-F5HP optimization for the 3-DoF case required approximately 12 min on a workstation equipped with an Intel Core i9-class processor running at approximately 5.0 GHz. This cost corresponds to the offline HP optimization stage only; once the champion HPs are obtained, the UKF can be run without repeating the NOMAD optimization for every subsequent identification scenario.

#### Joint state-parameter estimation

We use the optimal HPs in Table 2 to estimate the states and parameters of the system. We also use the same initial values for the quantities of interest (Eq. 21) in the UKFs throughout both HPs optimization and final identification of the system. We consider a known mass matrix, as well as zero initial displacement and velocity for all DoFs, with a covariance of one. The initial stiffness is initialized at 30 000 N/mm with a covariance of $10^7\mathbf{I}_n$, and the initial damping is set to 100 N s/m with a covariance of $10^4\mathbf{I}_n$.

Each variant of the UKF estimates the states and identifies the parameters, as illustrated in Fig. 2d–o. The states, shown in sub-figures (d–i), converge rapidly to the ground truth. To maintain clarity, we present only the second DoF states; the behavior of the other DoFs is similar. For the parameters, the filter estimation is shown with a different color for each DoF, as pointed out in Fig. 2j–o. The UKF-F5HP and UKF-FW7HP converge to the ground truth in approximately three seconds, whereas the UKF-W5HF requires about ten seconds to reach the ground truth. These results demonstrate the effectiveness of the UKF variants with the selected HPs in accurately estimating the system states and parameters, hence achieving the the first goal of this case study.

**Table 2:** The optimal HPs of the UKF with different adaptive schemes for the 3DoF damped mass spring system

Type of UKF	HPs	values
UKF-F5HP	$(r_0, q_0, \alpha _r, \alpha _q, \lambda )$	(0.0049, 1e-07, 0, 0.008, 1.3)
UKF-W5HP	$(r_0, q_0, w_q, w_r, \lambda$ )	(0.034, 0.306, 1500, 6200,1.6)
UKF-WF7HP	$(r_0, q_0, \alpha _r, \alpha _q, w_q, w_r, \lambda )$	(0.008, 0.07, 0.4, 0.45, 2405, 6999, 1.2)

#### Damage quantification

To evaluate the robustness of the optimal HPs under sudden parameter changes applied to the system and determine whether a new set of HPs is needed for each change in the system, a rapid alteration of the system’s physical properties is introduced at $t= 20\,{\hbox {s}}$ into the identification process. We reduce the stiffness to 45 000 N/m (10

Regarding the identified parameters, the UKF-F5HP exhibits difficulties in adapting to changes, particularly for estimating the damping values. Although the UKF-W5HP responds to the changes, it experiences delays and shows a greater uncertainty of estimation compared to the UKF-WF7HP, which demonstrates superior performance in tracking parameter variations accurately and with less uncertainty.

#### Uncertainty quantification

**Fig. 3:**
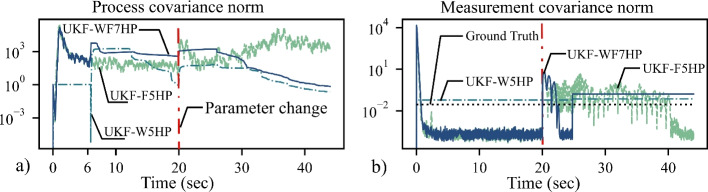
Temporal evolution of the norms of the process and measurement covariance matrices, respectively, for three variants of the adaptive UKF, presented in three different colors. Following the parameter change, the process error norm for UKF-W5HP and UKF-WF7HP decreases over time, reflecting the filters’ ability to converge to the ground truth. In contrast, the UKF-F5HP shows an increase in process error. In Figure **b**, both UKF-W5HP and UKF-WF7HP accurately capture the noise level imposed on the data (dotted black line), while UKF-F5HP fails to quantify the measurement error before or after the parameter change

We analyze the evolution of the covariance matrix norms for modeling uncertainties ($\mathbf{Q}$) and measurement uncertainties ($\mathbf{R}$) in different variants of the optimized adaptive UKFs over time, as shown in Fig. 3. In Figure **a**, the spike around the sixth second is explained by the window size of 1500 samples (6 *× * 250, with 250 as the data sampling rate) used in UKF-W5HP as shown in Table 2. The norm of process error in UKF-W5HP and UKF-WF7HP then exhibit a gradual and sustained decrease - particularly after the parameter change at *t= 20 s (red dashed line)* (red dashed line) – indicating effective identification of state parameters and progressive reduction in model-process mismatch. Because the true process noise level is not known a priori and may vary during identification, no fixed reference line is shown in Fig. 3a. In this setting, once the filter recovers the true physical parameters, the expected process noise naturally diminishes; the observed downward trend is therefore interpreted as successful adaptation rather than a failure to converge. By contrast, UKF-F5HP exhibits an increasing process covariance norm, indicating that it does not capture the parameter change and cannot reduce the process-model discrepancy.

Figure 3b presents the evolution of the measurement covariance norm, where both UKF-W5HP and UKF-WF7HP correctly estimate the measurement uncertainty and converge to the ground truth noise level (dotted black line) after the parameter change. In particular, UKF-W5HP quickly adapts to the imposed noise level with minimal fluctuations, while UKF-WF7HP shows a more gradual convergence with slightly higher variations. However, UKF-F5HP fails to capture the correct noise level, erroneously estimating a decreasing measurement uncertainty, which contradicts the actual constant noise level imposed on the data.

#### Robustness to non-Gaussian and time-varying noise

To further evaluate the robustness of the optimized HPs, the UKF-WF7HP is tested under additional noise scenarios beyond the Gaussian white-noise condition considered in the previous section. For this purpose, the optimal HPs obtained for the UKF-WF7HP are kept unchanged and are directly used in the new simulations. Therefore, the aim of this analysis is not to re-optimize the filter for each noise type, but rather to examine whether the already optimized HPs remain effective when the statistical nature of the noise changes.

Three noise scenarios are considered. The Gaussian case adds zero-mean normal response noise with standard deviation $10^{-2}$. The non-Gaussian case uses a zero-mean heavy-tailed Student-*t* normalized to unit variance, plus sparse Laplace impulsive outliers with a probability of *p=0.01* and scale 8; this distribution is symmetric, so its nominal skewness is zero, but its fourth moment/kurtosis is unbounded because of the $t_3$ tail (three degrees of freedom). The time-varying case uses the same non-Gaussian model but multiplies its scale by an intensity envelope

24 $$\begin{aligned} e(\tau )=0.35+1.15\tau +0.55\sin ^2(2\pi \tau ), \qquad \tau \in [0,1], \end{aligned}$$

where *τ * is normalized time; thus the noise standard deviation changes from about *0.35* to *1.78* times the base level, with variance scaling by $e^2(\tau )$. The magnitude of the added noise in the three cases is selected to be approximately comparable so that the comparison highlights the influence of the noise distribution and time variation rather than a simple increase in noise amplitude.

**Fig. 4:**
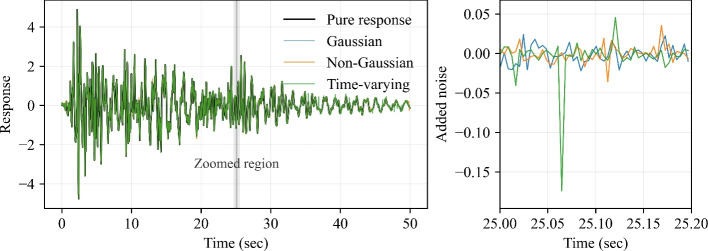
Added Gaussian, non-Gaussian, and time-varying noise used in the robustness analysis. The three noise cases are selected with comparable magnitude, while the non-Gaussian and time-varying cases introduce different statistical characteristics

**Fig. 5:**
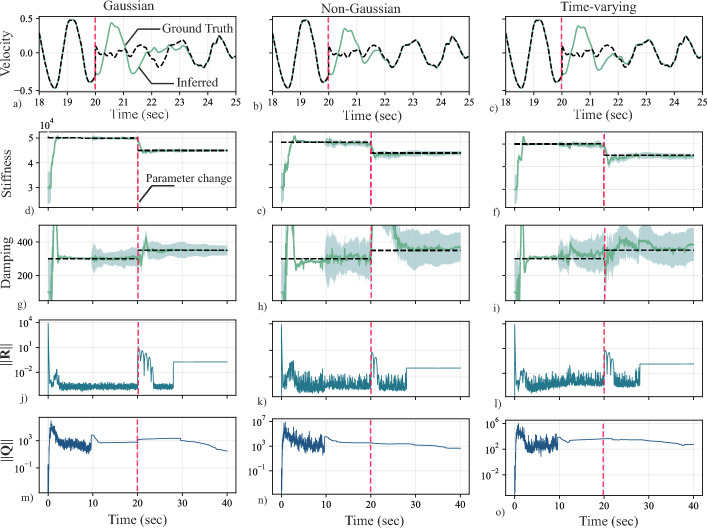
Robustness of the optimized UKF-WF7HP under Gaussian, non-Gaussian, and time-varying noise. The figure compares the estimated states, identified stiffness and damping parameters, and the evolution of the measurement and modeling uncertainty matrices, $\mathbf{R}$ and $\mathbf{Q}$, for the three noise scenarios

As shown in Fig. 4, the non-Gaussian and time-varying disturbances remain of the same order of magnitude as the Gaussian noise, while their statistical nature is different. This makes the test more representative of practical conditions in which the noise may not be independent, stationary, or exactly Gaussian.

Using the same optimized HPs, the UKF-WF7HP remains stable under these additional noise scenarios and preserves its ability to track the system response and parameter changes, as shown in 5. However, the results also show the expected effect of the additional uncertainty introduced by the non-Gaussian and time-varying noise. In the stiffness-estimation plots (Figs. 5(e) and 5(f)) and damping-estimation plots (Fig. 5h and i), the estimated parameters exhibit slightly stronger fluctuations than in the Gaussian-noise case. The uncertainty bounds also become wider, indicating that the filter detects the higher uncertainty level in the data rather than producing an overconfident estimate.

A similar trend is observed in the estimated measurement and modeling uncertainty. For the measurement-error evolution (Fig. 5k and l), the non-Gaussian and time-varying cases produce larger uncertainty levels because additional error is introduced at each time step. For the modeling-error evolution (Fig. 5n and o), the Gaussian-noise case shows a decreasing trend as the filter converges, whereas the non-Gaussian and time-varying cases remain almost constant. This behavior is consistent with the fact that the adaptive filter attributes part of the persistent non-Gaussian and time-varying disturbance to an effective modeling/process uncertainty.

Overall, these results indicate that the optimized adaptive algorithm is not only tracking the states and parameters, but also quantifying the additional uncertainty involved in the data. In other words, under more complex noise conditions, the filter responds by increasing the estimated uncertainty and maintaining stable parameter identification, instead of forcing the uncertainty to decrease artificially. This robustness is expected because the UKF-WF7HP updates both modeling and measurement covariance matrices through the combined moving-window and forgetting-factor adaptation, allowing the filter to adjust to changes in the effective uncertainty level during the identification process.

#### Discussion

In Fig. 2, the optimal HPs identified using the $L_2$ physics-aware objective function are shown to be effective for all three UKF variants. Notably, the UKF-F5HP rapidly aligns with the ground truth states but exhibits slower damage quantification compared to UKF-W5HP and UKF-WF7HP. This highlights that the ability of the UKF to track and quantify damage depends more on the chosen adaptive scheme than on HP tuning alone. Accordingly, window-based adaptive schemes are recommended for damage quantification, while forgetting factor-based schemes are better suited for fast joint state-parameter estimation tasks.

Another important point is the observed discrepancy between the filter’s performance in parameter estimation versus state estimation. This explains why an objective function based solely on the $L_2$ data-based norm (mean squared error between estimated outputs and sensor data) can be insufficient to properly tune the Bayesian solver. Since the states converge quickly to the ground truth, the loss decreases rapidly and flattens out, which can cause the optimization to stop exploring further improvements even though the parameters have not yet fully converged.

Furthermore, Fig. 3 highlights that window-based methods (UKF-W5HP and UKF-WF7HP) are a better option for quantifying modeling and measurement uncertainties compared to UKF-F5HP. Window-based approaches successfully mitigate process errors over time and reliably quantify measurement uncertainties, making them better suited to identify sudden changes during the identification process.

### Case study 2: identification of a ten-DoF system with 40 unknown states and parameters

In this case study, we extend a previous work on joint state-parameter estimation for a 10-DoF damped mass spring system excited by the El Centro earthquake time history [49]. Ghorbani and Cha [11, 29] attempted to identify the parameters and estimate the hidden states of the system using a standard UKF approach (UKF-3HP) assuming that all DoFs share identical stiffness and damping properties, but failed to adjust the UKF to capture the ground truth for the stiffness and damping values.

In this study, we tackle a more generalized and complex version of the same problem by imposing 5% noise on the measured acceleration data and introducing variable stiffness and damping properties across different DoFs. Furthermore, instead of UKF-3HP, we use adaptive UKF-F5HP with two more HPs to tune. As demonstrated in Fig. 2-j and stated in the discussion part of the Sect. 4.1, we selected the forgetting factor as the adaptive technique for this case study because UKF-F5HP quickly converges to the ground truth.

#### Problem formulation

The stiffness values for the first two DoFs are set to 40 000 N/m,, with an increment of 5000 N/m applied every two DoFs. This incremental pattern results in stiffness values for the 9th and 10th DoFs reaching 60 000 N/m.. The damping values are set to 500 N s/m for the first five DoFs and 400 N s/m for the remaining five DoFs. The mass for each DoF is uniformly set at 500 kg. The system is subjected to 40 s of El of El Centro earthquake excitation, serving as ambient vibration input.

Synthetic data, denoted as $\mathbf{y}$, are generated by adding 5% Gaussian noise to the acceleration responses specifically at the 1st ($\ddot{\mathbf{q}}_1$), 5th ($\ddot{\mathbf{q}}_5$), and 10th ($\ddot{\mathbf{q}}_{10}$) DoFs. This deliberate selection of a limited subset of observed sensors is intended to emulate a challenging identification scenario characterized by sparse sensor coverage and measurement noise. Such conditions represent an extreme case of partial observability, which is common in full-scale structural health monitoring practice. The aim is to evaluate the robustness and accuracy of the framework under these realistic and difficult constraints where only a fraction of the system states are directly measured, thereby testing the capability of the tuning approach and filtering method to infer unmeasured states and parameters reliably.

The augmented state vector has a size of 40 consisting of one displacement, velocity, stiffness, and damping value for each DoF:

25 $$\begin{aligned} \mathbf{x}_{40 \times 1} = \left( q_1, \dots , q_{10},\; \dot{q}_1, \dots , \dot{q}_{10},\; k_1, \dots , k_{10},\; c_1, \dots , c_{10}\right) ^\top . \end{aligned}$$

In the identification process, the displacement and velocity of the vibration are initially set to zero with a covariance of one. The stiffness values for all DoFs are set to 300 N s/m, with a covariance of $10^9$, while the damping values are set to 30 000 N/m, with a covariance of $10^5$. Next, the UKF-F5HP is integrated into the NOMAD optimization algorithm (see Fig. 1) to determine the optimal set of HPs for system identification. Assuming the scaling factor * λ * is set to zero, the initial HPs are arbitrarily set to $(r_0, q_0, \alpha _r, \alpha _q) = (0.01, 6 \times 10^{-3}, 1, 0.9)$. After 400 iterations, NOMAD optimizes the HPs to $(r_0, q_0, \alpha _r, \alpha _q) = (0.26, 10^{-7}, 0, 0.2)$. The values $\alpha _r = 0$ and $\alpha _q = 0.2$ indicate that NOMAD selected a fixed measurement error and a gradually decreasing process error throughout the identification process. The UKF-F5HP optimization required approximately 20 min on a workstation equipped with an Intel Core i9-class processor running at approximately 5.0 GHz.

**Fig. 6:**
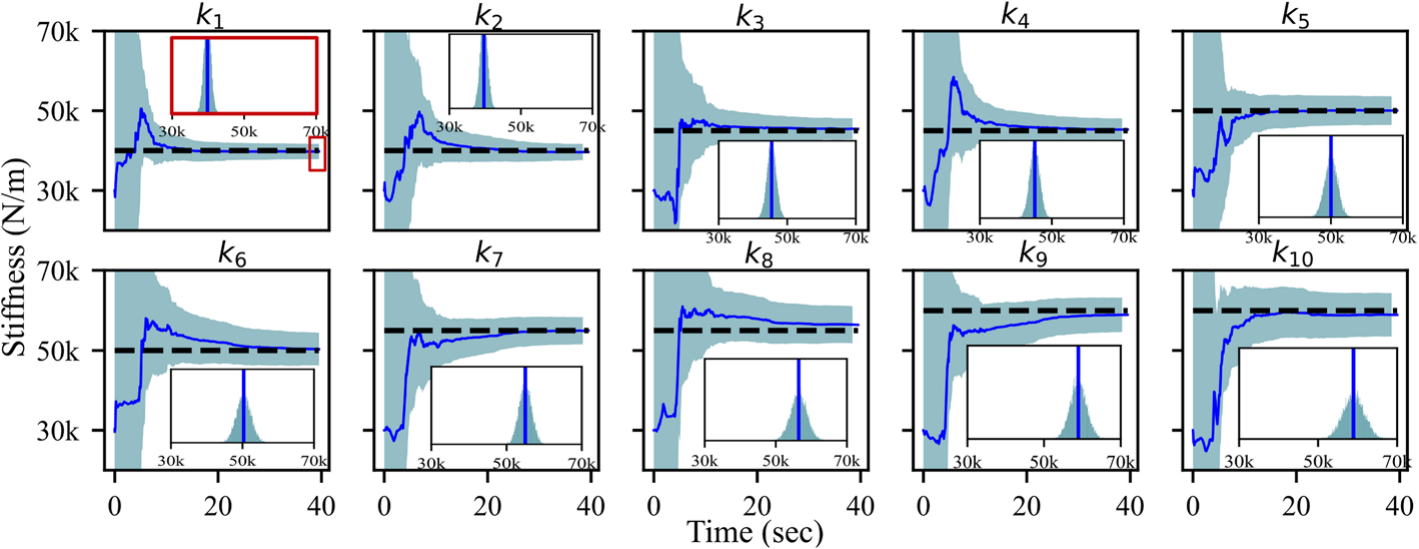
Time evolution of the estimated stiffness matrix terms corresponding to the ten DoF system: UKF-F5HP estimation (blue solid line); estimation uncertainty (grey area); ground truth values (black dashed line). Inset show the histogram of the last time step estimated parameter for each DoF, taking uncertainty into account. Both the estimation uncertainty and the convergence time increase for higher DoFs, due to the widening gap between prior information and the actual values

For brevity, the evolution of the physics-aware objective function using the NOMAD algorithm is not shown, as well as the estimated displacement and velocity of vibration. Instead, we focus on presenting the estimated parameters obtained using the optimal set of HPs.

#### Results and discussion

Figure 6 illustrates the evolution in time of the estimated stiffness matrix, where all stiffness values were initially set to 30 000 N/m. The results indicate that the filter successfully estimates the ground truth, depicted by the blue line, while the shaded region represents the estimation uncertainty across all DoFs.

For each stiffness term, the inset provides a histogram of the estimated mean and variance at the last time step of the identification process. As shown, the uncertainty increases for higher DoF stiffness values, likely due to the identical initial values assigned to all DoFs. In particular, for $k_{10}$, where the ground truth stiffness value is nearly twice the initial value, the filter accurately estimates the stiffness despite the substantial initial discrepancy.

**Fig. 7:**
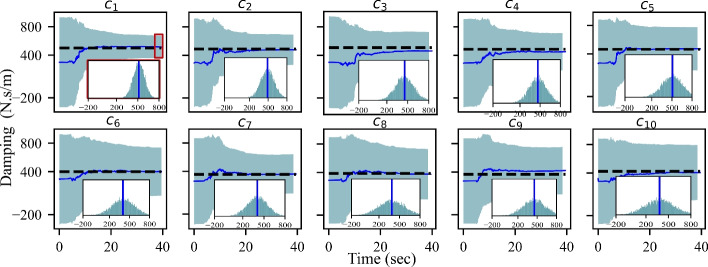
Time evolution of the estimated damping matrix terms corresponding to the ten DoF system: UKF-F5HP estimation (blue solid line); estimation uncertainty (grey area); ground truth values (black dashed line). Inset show the histogram of the last time step estimated parameter for each DoF, taking uncertainty into account

The damping matrix is illustrated in Fig. 7. The tuned adaptive UKF accurately estimated the damping values for all DoFs, as shown by the blue lines. As expected, the uncertainty in the damping estimation is greater than in the stiffness estimation, as shown with the shadow area in both the time evolution and the histogram of the latest estimated mean and variance [50].

The estimated displacement and velocity of the vibration, which are not shown here, also converge rapidly to the true values. A comparison with the results presented by [11] demonstrates that the proposed automated tuning approach for the UKF effectively addresses the challenges of identifying parameters in dynamical systems with many DoFs [51]. This physics-aware framework surpasses the limitations of the traditional hand-tuned UKF method by accurately estimating system states and parameters, even when assigning different stiffness and damping values for each DoF and utilizing parial observations only from the 1st, 5th, and 10th DoFs. These results highlight the robustness and efficiency of the automated tuning process in handling complex and multi-DoF dynamical systems.

The larger uncertainty observed in the damping estimates compared with the stiffness estimates also reflects the practical identifiability of this ten-DoF problem. Although the optimized UKF converges to the correct parameter range, the damping parameters are less strongly informed by the available sparse acceleration measurements than the stiffness parameters; therefore, their posterior spread remains larger. This observation should be distinguished from the accuracy of the estimated displacement and velocity states. Because the Kalman correction step directly uses the measured outputs, the filter can accurately track the observed dynamic response while some latent physical parameters remain weakly constrained.

This behavior illustrates a still open question in the physics-aware HP optimization. The proposed objective improves HP tuning by favoring estimated trajectories that satisfy the governing equations, but it does not remove the identifiability requirements of the underlying inverse problem. When the augmented state-space model is weakly identifiable over the available sensing and excitation window, different combinations of stiffness, damping, process noise, and measurement noise may produce similar measured outputs and comparable physics residuals. Consequently, the physics-aware loss landscape may contain flat valleys or several near-equivalent local minima, and different HP sets may lead to similar values of $\mathcal {L}^{2}_{\text {physics}}$ while producing different parameter trajectories along weakly observable directions.

The present case study was intentionally designed as a stringent robustness test: all stiffness and damping parameters were assigned different ground-truth values but initialized from the same starting guess, with some true values lying as far as 100 % away from the initial estimate. The ability of the proposed physics-aware tuning to drive the parameters toward their distinct target ranges under sparse sensing highlights the robustness of the method. At the same time, the wider damping uncertainty indicates that the results should be interpreted in an uncertainty-aware manner rather than as equally identifiable estimates for all parameters.

One way to address this issue in applications with sparse sensors, many DoFs, correlated parameters, or abrupt physical changes is to compare multi-start NOMAD runs not only by their final physics-aware loss but also by the consistency of the recovered parameter trajectories. If several HP sets produce comparable loss values but different parameters, the corresponding parameters should be reported as weakly identifiable rather than as unique estimates. Practical identifiability can be improved by using sensitivity, observability, or Fisher-information-type analyses before optimization, enforcing physically admissible bounds and scaling during optimization, reducing the number of simultaneously estimated parameters when needed, and, when possible, increasing excitation richness or adding strategically placed sensors.

### Case study 3: comparison of objective functions

In this section, we take a closer look at the physics-aware objective function and compare its effectiveness with traditional data-based objective functions. The four objective functions to find the optimal HPs of the UKF considered in this study are

- $L_2$ norm physics-aware objective function:
  26 $$\begin{aligned} \mathcal {L}^2_\text {physics} =\sqrt{ \sum _{k=k_1}^{k_2} \left( \hat{\mathbf{x}}_{k+1} - \mathcal {M}(\hat{\mathbf{x}}_k, \mathbf{u}_k, \boldsymbol{\theta }) \right) ^2 }. \end{aligned}$$
- $L_2$ norm data-based objective function:
  27 $$\begin{aligned} \mathcal {L}^2_{\text {data}} = \sqrt{\sum _{k=k_1}^{k_2} \left( \mathbf{y}_k - \hat{\mathbf{y}}_k \right) ^2}. \end{aligned}$$
- $L_1$ norm physics-aware objective function:
  28 $$\begin{aligned} \mathcal {L}^1_\text {physics} = \sum _{k=k_1}^{k_2} \left| \hat{\mathbf{x}}_{k+1} - \mathcal {M}(\hat{\mathbf{x}}_k, \mathbf{u}_k, \boldsymbol{\theta }) \right| . \end{aligned}$$
- $L_1$ norm data-based objective function:
  29 $$\begin{aligned} \mathcal {L}^1_{\text {data}} = \sum _{k=k_1}^{k_2} \left| \mathbf{y}_k - \hat{\mathbf{y}}_k \right| . \end{aligned}$$

#### Problem formulation

The Lorenz system is selected because it serves as a canonical example of a chaotic nonlinear system, characterized by extreme sensitivity to initial conditions and parameters. Based on the work of [52], the Lorenz system provides a challenging yet well-studied benchmark to evaluate the capability of a system identification algorithm with partial and noisy observations. Their results showed that any subset of the Lorenz parameters can be recovered provided the corresponding state variables are observed, including scenarios where one state component is completely unobserved. This highlights the Lorenz system’s suitability for testing the robustness and effectiveness of the optimized adaptive UKF and the physics-aware objective function under conditions of partial observation and noise [53]. The Lorenz system equations are:

30 $$\begin{aligned} {\left\{ \begin{array}{ll} \dot{q}_x & = \sigma (q_y - q_x), \\ \\ \dot{q}_y & = q_x(\rho - q_z) - q_y, \\ \\ \dot{q}_z & = q_x q_y - \beta q_z, \end{array}\right. } \end{aligned}$$

wherein for certain values of parameters, like *σ = 10*, *ρ = 28*, and *β = 8/3*, the system exhibits chaotic behavior [54].

The system is simulated for 50 s at a sampling rate of 300 Hz. To emulate a partial observation scenario during the identification process, only vibration displacements in the *x* and *z* directions are considered as sensor measurements, denoted as $\mathbf{y} = (\mathbf{q}_x, \mathbf{q}_z),$ for joint state-parameter estimation. Furthermore, white noise with an SNR of 15 is added to the simulated data to enhance the generality of the identification task.

The augmented state vector consists of six states and three parameters,

31 $$\begin{aligned} \mathbf{x} = \left( q_x, q_y, q_z, \dot{q}_x, \dot{q}_y, \dot{q}_z, \sigma , \rho , \beta \right) ^\top = \left( x_1, x_2, x_3, x_4, x_5, x_6,x_7, x_8, x_9 \right) ^\top , \end{aligned}$$

which leads to the state transition function as:

32 $$\begin{aligned} \mathcal {M}_c^{\text {Lorenz}}(\mathbf{x}_k, \mathbf{u}_k) = \begin{bmatrix} x_4 \\ \\ x_5 \\ \\ x_6 \\ \\ x_7 (x_2-x_1) \\ \\ x_1(x_8-x_3)-x_2 \\ \\ x_1 x_2-x_9x_3\\ \\ \mathbf{0}_{3 \times 1} \\ \end{bmatrix}, \end{aligned}$$

where the subscript $\text {c}$ stands for continuous function. Following that, the measurement function for the partial observation scenario is defined as:

33 $$\begin{aligned} \mathcal {H}^{\text {Lorenz}}(\mathbf{x}_{k}, \mathbf{u}_k)= \left[ x_1 \quad x_3 \right] ^\top = \left[ q_x \quad q_z \right] ^\top . \end{aligned}$$

The four objective functions (Eqs. 26–29) are used to find the optimal HPs of UKF-F5HP. All four optimization processes start with the same initial HPs values, as well as same initial values for the augmented state vector in Eq. (31). The filter is optimized using the four objective functions over 200 iterations and the optimal HP is used for identification of the Lorenz system as well as comparison of effectiveness of the objective function.

#### Joint state and parameter estimation under partial observation

**Fig. 8:**
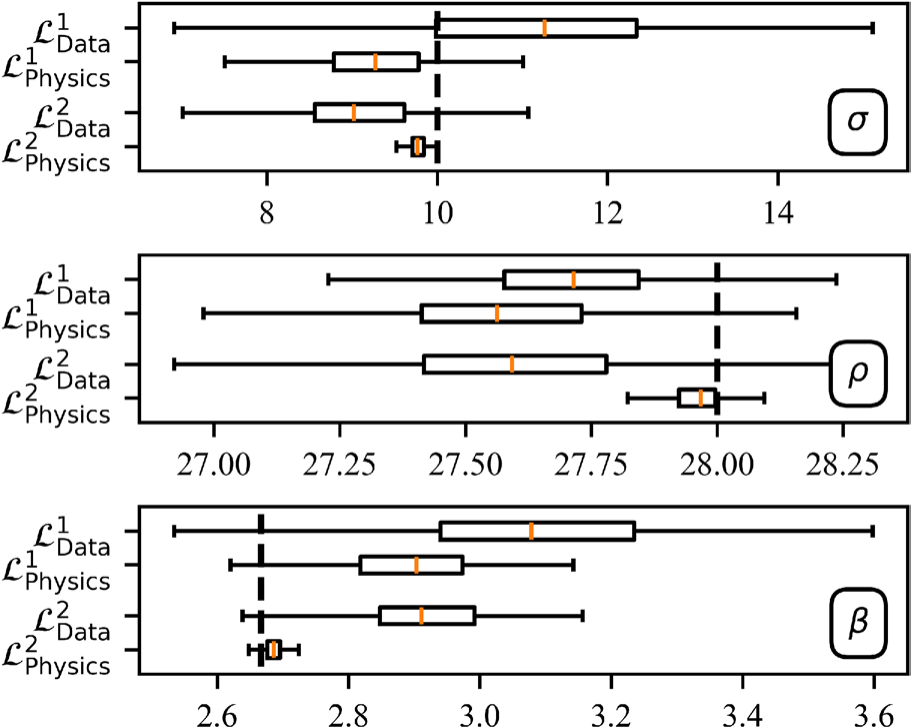
The identified parameters by the UKF-F5HP at the final time step, using the optimal HPs derived from four different objective functions. The orange line represents the median value and the whiskers indicate the range of variation. As shown, the physics-aware objective function based on the $L_2$ norm provides the most accurate and consistent parameter estimations across all cases

Figure 8 illustrates the latest parameter estimations of the optimal UKF based on different objective functions. The box plots present the estimated parameters of the Lorenz system * σ *, * ρ *, and * β *, using four different objective functions. Each box plot provides a visual representation of the estimation distribution, with the orange line indicating the median value and the whiskers showing the range of variation.

For the parameter * σ *, it can be observed that the $L_2$ norm physics-aware objective function yields the most concentrated estimates with the least uncertainty, while the data-based approaches exhibit larger variance and potential bias. Similarly, for the parameter * ρ *, the physics-aware objective functions demonstrate tighter distributions compared to the data-based methods, highlighting their effectiveness in improving parameter accuracy. In the case of * β *, the physics-aware methods again show superior performance with lower variability, while the data-based methods introduce a wider range of estimates.

Overall, the results suggest that the physics-aware objective functions, particularly those based on the $L_2$ norm, provide more stable and accurate parameter estimations compared to their data-based counterparts. This highlights the advantage of incorporating physical knowledge into the optimization process to improve the robustness of the parameter identification in chaotic systems like the Lorenz system.

#### Why physics-aware loss function

**Fig. 9:**
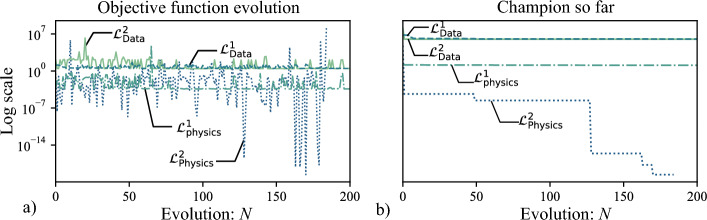
The efficacy of four objective functions in HP optimization for the Lorenz system. Figure **a** demonstrates the $L_2$ norm physics-aware objective function shows the most significant reduction, indicating its superior performance in capturing the system dynamics and optimizing the HPs. Figure **b** shows the local minima within the optimization process, referred to as the “champion so far" solutions, are shown for each objective function. The flat lines represent periods with no improvements, while the downward steps indicate progress in achieving better objective values

Figure 9 presents the evolution of the four objective functions used to optimize the HPs for the Lorenz system. Figure 9-a shows the progression of the objective function values over the course of 200 iterations. The logarithmic scale highlights the differences in convergence rates and magnitudes across the four objective functions. Figure 9-b provides a stepwise representation of the best objective function values found throughout the optimization process, referred to as the *champion so far*. The flat lines in this figure represent periods during which no improvement in the objective function was achieved, whereas the downward steps indicate instances where the optimization algorithm identified a new, lower objective value. The figure highlights that the $L_2$ norm of physics-aware objective function consistently reaches lower values compared to the other objectives, reaffirming its effectiveness in optimizing HPs.

The superior performance of the $L_2$ norm of the physics-aware objective function compared to the data-based objective function can be explained by analyzing the results presented in Fig. 2 in Sect. 4.1. A detailed comparison of the estimated states and parameters in these figures reveals that following a sudden change in the system parameters at * t= 20\,s *, the estimated states quickly converge to their true values, as shown in Fig. 2 (sub-figures d–i). However, the parameter estimation process takes significantly longer to stabilize, as illustrated in Fig. 2 (sub-figures j–o). The data-based objective functions, which rely solely on comparing the estimated states with sensor measurements, may incorrectly assume that the system has fully converged to the ground truth once the states align with the measurements. Consequently, they may prematurely stop the search process under the false impression that the optimal HPs have been found. In reality, further optimization is required to accurately identify the system parameters.

In contrast, the physics-aware objective function, which incorporates both the estimated states and parameters within the governing equations of the system, is more effective in capturing the underlying physics. This prompts the filter and optimization algorithm to continue searching for improved HPs until the parameters are accurately identified, minimizing the physical equation. This explains the effectiveness of the physics-aware loss function in finding the optimal HPs for joint state-parameter estimation, partial observations, and systems with many DoFs rather than data-based loss functions.

**Fig. 10:**
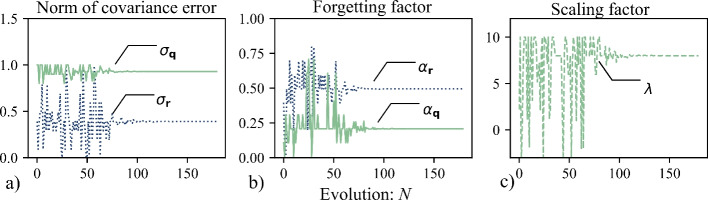
Progression of HPs throughout the NOMAD optimization process for the $L_2$ norm physics-aware objective function. Figure **a** illustrates the evolution for the initial values of the process and measurement covariance matrices, **b** for their forgetting factors and **c** shows the evolution of the scaling factor of the sigma points. The figure highlights the selected range for each HP and the number of iterations required for the optimization algorithm to reach stability

#### Search space of NOMAD optimizer

**Fig. 11:**
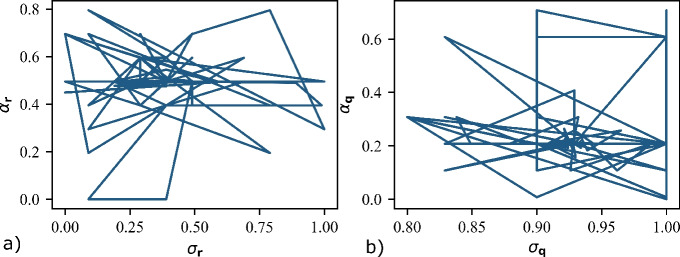
The search space of HPs during the NOMAD optimization evolution for the $L_2$ norm physics-aware objective function. Each line represents the trajectory of HP values explored across iterations for a specific optimization process. **a** depicts the relationship between $\sigma _r$ and $\alpha _r$, **b** presents the relationship between $\sigma _q$ and $\alpha _q$, highlighting the trajectory of the search in this parameter space

Figures 10 and 11 illustrate the evolution of five HPs for the physics-aware objective function in two different plots. The parameters $r_0$ and $q_0$ represent the initial process and measurement covariance values, while *α * denotes the forgetting factor. The parameter *λ * is associated with the UT scaling factor. Figure 10 shows that all HPs converge to stable values after several iterations, indicating the optimal configuration, while Fig. 11 displays the HP search space across the predefined ranges. The figure shows certain regions that the NOMAD algorithm did not explore, suggesting potential opportunities to enhance the framework’s performance through further optimization in these unexplored areas.

As a derivative-free optimizer, NOMAD naturally relies on user-defined elements such as initial mesh size, polling directions, and bound settings, and its search path can be influenced by these choices. In the present study, we employed reasonable initial values and observed consistent convergence of the hyperparameters, suggesting that the identified optima are robust to typical initialization choices. A comprehensive analysis of NOMAD parameter sensitivity, however, lies outside the scope of this work, whose primary goal is to demonstrate the capabilities of the physics-aware objective function. Future investigations could explore advanced configurations–such as multi-start strategies or adaptive adjustment of NOMAD parameters–to further quantify and potentially accelerate convergence.

## Conclusion

This study extends the use of a physics-aware objective function for automated HP tuning of UKF-based joint state-parameter estimation. In the first case study, the optimized adaptive UKF variants successfully tracked abrupt parameter changes, quantified uncertainty, and remained robust under different noise conditions, including non-Gaussian and time-varying noise. These results show that the choice of adaptive scheme is important for reliable change quantification. In the second case study, the proposed UKF-NOMAD framework identified stiffness and damping parameters in a ten-DoF system with 40 unknown states and parameters under sparse and noisy measurements, reducing the reliance on manual trial-and-error tuning for high-dimensional dynamical systems with limited observations. In the third case study, the results showed why the physics-aware objective outperformed conventional data-based objectives: it selected HPs that better enforce consistency with the governing equations rather than only fitting the measured outputs.

Future work will extend the framework to weak-form physics residuals, hybrid physics-data objectives with adaptive loss weighting, and probabilistic optimization strategies for UKF HP tuning. These extensions will be assessed with respect to identification accuracy, computational cost, and optimization complexity.

Overall, the proposed physics-aware UKF-NOMAD framework provides a practical tool for digital twins of mechanical and structural systems. Its ability to support state estimation, damage quantification, anomaly detection, and uncertainty quantification can help enable intelligent maintenance strategies and remaining useful life prediction for renewable energy systems and civil infrastructure.

## Data Availability

The data supporting the findings of this study were generated by numerical simulations described in the manuscript. No experimental data were used. The simulation inputs and procedures are fully detailed in the paper, and all results can be reproduced from the described methodology. Due to confidentiality agreements with industrial partners, the underlying simulation codes are not publicly available; however, they can be made available by the corresponding author upon reasonable request.
